# Ruminal Phages – A Review

**DOI:** 10.3389/fmicb.2021.763416

**Published:** 2021-12-08

**Authors:** Richard R. Lobo, Antonio P. Faciola

**Affiliations:** Department of Animal Sciences, University of Florida, Gainesville, FL, United States

**Keywords:** microbiome, phage, ruminal ecology, ruminant nutrition, virome

## Abstract

The rumen ecosystem is a complex and dynamic environment, which hosts microorganisms including archaea, bacteria, protozoa, fungi, and viruses. These microorganisms interact with each other, altering the ruminal environment and substrates that will be available for the host digestion and metabolism. Viruses can infect the host and other microorganisms, which can drive changes in microorganisms’ lysis rate, substrate availability, nutrient recycling, and population structure. The lysis of ruminal microorganisms’ cells by viruses can release enzymes that enhance feedstuff fermentation, which may increase dietary nutrient utilization and feed efficiency. However, negative effects associated to viruses in the gastrointestinal tract have also been reported, in some cases, disrupting the dynamic stability of the ruminal microbiome, which can result in gastrointestinal dysfunctions. Therefore, the objective of this review is to summarize the current knowledge on ruminal virome, their interaction with other components of the microbiome and the effects on animal nutrition.

## Graphical Abstract

Ruminal viruses can interact with all groups of microorganisms in the ruminal environment, which include bacteria, archaea, fungi, and protozoa. Physical-chemical parameters of the ruminal environment, such as pH, concentration of volatile fatty acids (VFA), ammonia, and other chemical components, can be modulated by the animal’s diet. We hypothesized that such changes in the physical-chemical parameters of the ruminal environment can modify the infectivity of viral particles and change the lysis rate of microbial cells, consequently, changes on VFA production and microbial biomass could affect animals’ performance.

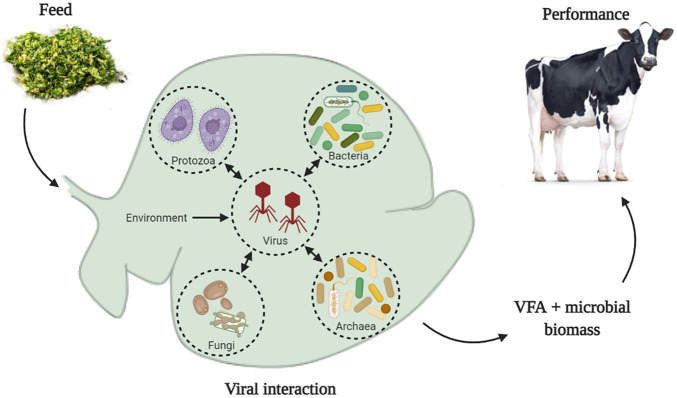

## Introduction

The rumen is a complex and dynamic ecosystem inhabited by anaerobic bacteria, protozoa, fungi, archaea and viruses (Huws et al., 2018), all living in the same environment and interacting with each other. This microbial community uses a wide range of feed components as substrates for its own growth, including cellulose- and starch-rich substrates. In a healthy ruminant this process generates volatile fatty acids which are absorbed by the ruminal epithelium and used as the major energy source (approximately 70%) for the host animal (Bergman, 1990). Microbial growth also supplies microbial biomass which, given its protein concentration, amino acid profile, and digestibility, represent an important source of metabolizable protein for the host (Schwab and Broderick, 2017).

Ruminal bacteria, archaea, protozoa, and, to a less extent, fungi have been vastly studied in the past decades; however, ruminal viruses have mostly been under investigated but have been gaining more attention due to their biotechnological potential and possible effects on animal health and production. The most known description of viruses is that they are simple infectious particles composed of genetic material (DNA or RNA in a single or double strand) and an outer shell composed of protein, this simple structure is called a virion or free-virus (Lodish et al., 2000), also some viruses have an outer membrane and are called enveloped viruses (Tsai, 2007). The viruses population in the environment is referred to as the virome (Ross et al., 2013). Those viruses that specifically target and infect bacteria are called bacteriophages (phages) (Clokie et al., 2011). There are also viruses that infect other prokaryotes in the rumen such as archaea which may also be called archaeaphages or archaeal viruses, and eukaryotes such as fungi (called mycophages or mycoviruses) and protozoa (protozoan viruses) (Gilbert et al., 2020). Phages, particularly infecting bacteria, have been reported to be the most numerous virus population in the rumen, as well as the most studied, and will therefore be the focal point of this review.

The first description of these submicroscopic agents in the ruminal environment occurred in the 1960s when it was demonstrated that viruses were inhabitants of the rumen and not just transient of the gastro-intestinal tract (GIT), from feed and water ingested (Adams et al., 1966; Hoogenraad et al., 1967). These resident viruses use ruminal microorganisms to proliferate themselves (Gilbert and Klieve, 2015) and represent a significant part of the ruminal microbiome. The viral particle count reported in the literature ranges from 5 × 10^7^ to 1.4 × 10^10^ (Table 1).

**TABLE 1:** Summary of published electron microscopy (EM) and pulse-field gel electrophoresis (PFGE) studies characterizing the phage population in the ruminal environment.

Phage particle count^a^	Method used	Morphology types	Host species	Number of animals	Feed used	Source
NA	EM	NA	Sheep	NA	Lucerne chaff	Hoogenraad et al. (1967)
5 × 10^7^	EM	6	Cattle	1	Alfalfa hay	Paynter et al. (1969)
NA	EM	NA	Reindeer	NA	NA	Tarakanov (1972)
2 × 10^7^ to 1 × 10^8^	EM	26	Cattle/sheep	9	Chaffed rice straw and oaten chaff	Klieve and Bauchop (1988)
1.4 × 10^10^	PFGE	NA	Sheep	1	Oaten chaff and lucerne chaff	Klieve and Swain (1993)

*^a^Estimation of free phages total count.*

*NA – Non-available.*

Aside from resident viruses, transient viruses from feed and water sources can also be found in the rumen, which would likely make up a small part of the ruminal virome; however, they are not well understood and very little is known about their interaction with the ruminal microbiome. According to Klieve et al. (1996), the majority of the resident phages in the rumen have a symbiotic relationship with other ruminal microorganisms, and are often found integrated in host genomes as prophages. This interaction can create a long term and symbiotic association with the host cells, where the genome of the phage is inserted into the chromosome of the host organism and this process ends up forming the prophage. However, a predation interaction can also take place, where infection of the host cell by phages occurs with a short association, rapid death of the host cell, and release of new virions. These interactions between phages and microorganisms can drive changes in the microbial ecology of the environment (Koskella and Brockhurst, 2014). Those changes can modify ruminal fermentation and affect health of the animals, and consequently, animal performance (Namonyo et al., 2018) and will be covered in more details later on in the article.

Because of the limited knowledge about the ruminal virome and its interaction with other ruminal microorganisms, the objective of this review is to summarize the knowledge available in the literature in ruminal phages and create a comprehensive review, including the viral biology, their interaction with other microorganisms in complex environments, and their implication in the ruminant nutrition and health.

## Replication Cycle

**FIGURE 1:**
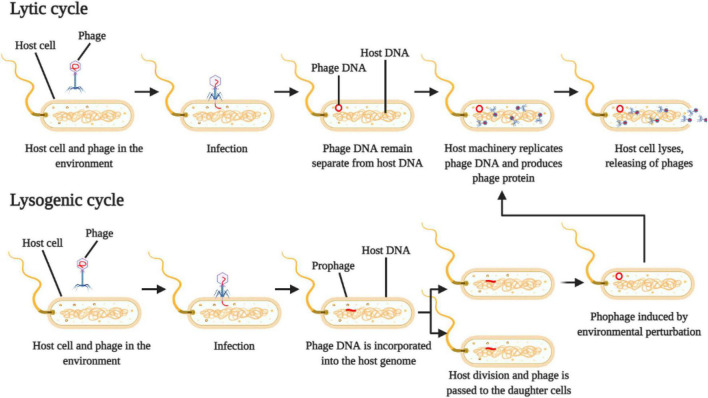
Schematic representation of phage reproduction by lytic and lysogenic cycle. Adapted from Payet and Suttle (2013).

The lifecycle of the phage is used to classify them into virulent (or strict lytic phages) and temperate phages (Figure 1). Virulent ones use the lytic cycle, which inject their own genetic material into the host cell and quickly reprogram the cell machinery to replicate the viral particles and transcribe viral proteins, leading to a fast death of the host and consequently release of new virions in the environment (Payet and Suttle, 2013).

On the other hand, temperate phages use the lysogenic cycle, which will inject their genetic material into the host cell, where the viral genome is incorporated into the host cell genome, creating the prophage (Payet and Suttle, 2013) (Figure 1). The host cell (lysogen) then replicates itself, and the resulting daughter cells both harbor the prophage integrated in their genomes. This prophage can stay in a dormancy stage for several generations and can be activated or induced by physical or chemical environmental perturbations, which will start to produce viral particles and eventually cell lysis (Paul, 2008).

Viral infection is highly specific and host cell receptors may be composed of lipopolysaccharide, sugars, proteins, and fimbriae structures, which will determine the range of organisms that can host the virus (Matsuzaki et al., 2005). Some phages only target and infect one host species or even strain, and may be classes as monovalent, whilst others can infect multiple strains and be classed as polyvalent (Parasion et al., 2014).

Phages are commonly associated with their destructive effects on the host cell; however, injection of genetic material into the host cell can actually guide it through a process of evolution and diversification via horizontal gene transfer (HGT) (Berg Miller et al., 2012; Koskella and Brockhurst, 2014; Gilbert et al., 2020). Horizontal gene transfer can occur in both prokaryotic and eukaryotic cells by a process called transduction, where a newly formed virus incorporate host genes and transfers it to another cell (Touchon et al., 2017). However, these genes usually have to improve the adaptation chances of the recipient cell or it will no longer survive in the new lineage formed (Soucy et al., 2015).

Prokaryotic cells can use a strategy of their adaptive immune elements to incorporate the foreign genetic material into their own genome via clustered regularly interspaced short palindromic repeats (CRISPR) and CRISPR associated (Cas) protein (Barrangou et al., 2007; Berg Miller et al., 2012; Barrangou and Marraffini, 2014). The CRISPR/Cas process occurs in a large number of species of archaea and almost all species of bacteria in the rumen and horizontal gene transfer is a common and important evolutionary practice of the living organisms in the ruminal environment (Berg Miller et al., 2012). The presence of CRISPR/Cas genes can provide historic information of phage-bacteria interaction and metagenomic analysis of the rumen microbiome (Berg Miller et al., 2012) and genomic analysis of bacterial host have detected CRISPR/Cas genes (Gilbert et al., 2017; Friedersdorff et al., 2020) suggesting previous interactions between phages and bacterial host.

## Key Groups of the Rumen Virome

Viruses that infect bacteria and those that infect archaea are often presented together due to its similarity (Gilbert and Klieve, 2015). A pioneer study of the characterization of ruminal phages using electron microscopy (EM) observed six different morphological viral types in the bovine ruminal content (Paynter et al., 1969), which is a small number when compared to the 26-40 morphological types observed by Ritchie et al. (1970) and Klieve and Bauchop (1988). Using molecular approaches (metagenomic) to identify phages and prophages in the ruminal environment, Berg Miller et al. (2012) and Anderson et al. (2017) identified a large number of viral DNA fragments in a large number of viral species (1500 and 2243, respectively), those DNA sequences were assessed for homology to the viral genome database, and the majority of them could not be identified, meaning that the majority of the phages and prophages in the ruminal environment were unknown.

Both, Berg Miller et al. (2012) and Anderson et al. (2017), observed that viruses (phages and prophages) from the families *Myoviridae*, *Siphoviridae*, *Mimiviridae*, and *Podoviridae* were the most abundant in the ruminal environment, their results corroborates the findings on morphological studies by Ritchie et al. (1970). According to Klieve et al. (1996), the majority of the phages in the ruminal environment are in a lysogenic state (prophages), which represents a symbiotic coexistence between phages and other microorganisms. Corroborating these results, Berg Miller et al. (2012) reported that prophages outnumbered lytic phages approximately 2:1 and the majority of the viruses (both prophages and phages) are proportionally associated with the dominant ruminal bacterial phyla (*Firmicutes* and *Proteobacteria*).

Culture-based studies have been used to isolate and culture new phages. Gilbert et al. (2017) reported the genomic study of five isolated phages of the families *Siphoviridae* and *Podoviridae*. Isolated phages were described as predators of ruminal bacteria of the genera *Bacteroides*, *Ruminococcus*, and *Streptococcus* and a co-examination of bacterial genomes suggests that these microorganisms have genes responsible for modulating phage:host interactions, such as CRISPR/Cas elements and restriction-modification phage defense. However, the authors suggested that even bacterial strains within the same genus could have different receptivity to phage infection and replication than others.

Another recent study reported five more phage genomes isolated from the ruminal environment, which double the currently available phage genomes (Friedersdorff et al., 2020). These phages have been observed in a lytic lifestyle, as a free phage; however, genomic analysis indicates that some have a potential to undergo temperate lifecycle. Also, the authors reported that the five phages identified were isolated from a single host (*Butyrivibrio fibrisolvens*) and they belong to three different genera, which indicate that there is much to be discovered and an international effort is required to achieve a greater understanding of the viral diversity and interaction.

Only a few studies on ruminal viruses that interact with prokaryotic organisms are available in the literature; however, fewer report viruses with eukaryotic (i.e., anaerobic fungi and protozoa) interactions. Anaerobic fungi is an important component of the microbial community in the ruminal environment, playing a key role in the degradation of plant cell wall material (Rabee et al., 2019; Azad et al., 2020). While the importance of anaerobic fungi in ruminant nutrition is known, very little is known about the mycoviruses that infect them. To our knowledge, only one study reporting mycoviruses in the rumen is available (Hitch et al., 2019). The authors reported the presence of 30 mycoviruses in the ruminal environment, which the majority were classified in the *Partitiviridae*, *Alphaflexiviridae*, and *Betaflexiviridae* families; however, similar to the bacteriophage analysis, the majority of the DNA sequences obtained could not be classified (only 0.025% of the contigs were related to known mycoviruses), indicating that more studies are needed to access a better identification of mycoviruses.

Anderson et al. (2017), in their metagenomic analysis of viruses, observed ten DNA fragments that belong to viruses that have eukaryotic cells as target hosts (fungi and protozoa) were identified, based on the homology with known viruses; however, no further explanations were provided. To the best of our knowledge, this is the only study evaluating ruminal protozoa viruses. However, it has been documented that viruses can infect protozoa in other environments (Barrow et al., 2020), suggesting that ruminal protozoa can also be infected by viruses, leading to speculations that protozoal population changes can be driven by viral activity, with possible implications in the fiber and protein utilization.

## Viral Influence in Complex Environments

As mentioned previously, phages are predators of other organisms. In a complex environment, such as the marine environment, viruses can be considered drivers of nutrient and energy cycles (Suttle, 2007). Phages can modulate bacterial populations through host cell lysis, which could in turn effect ruminant nutrition. Although this hypothesis has not been fully proven, we know from other microbiomes that virus can be considered drivers of nutrient and energy cycles (Suttle, 2007).

According to Mojica and Brussaard (2014), after the release of viral progeny, free virions are exposed to the environmental physicochemical conditions of the water (such as pH, temperature, and metabolites presented in the water) which can affect the interaction of the viruses with other organisms. For example, environmental conditions can change infectivity, modify the viruses’ structure, and adsorption of it by the host organism. Anthropogenic activities in the marine environment can change physicochemical conditions and metabolites composition of the water, these changes have impacts in the virus-host interaction and consequently on rate of host lysis (Mojica and Brussaard, 2014; Johannessen et al., 2017); however, the exact way that environmental properties affect the viruses are still unknown.

In addition to marine environments, the gut virome in humans has been well studied (Minot et al., 2011; Beller and Matthijnssens, 2019). Published studies reported that the human gut virome is driven by diet (Minot et al., 2011; Oh et al., 2019; Rasmussen et al., 2019). However, there is little explanation of how viral population structure is driven by diet and how it can affect other organisms within the GIT.

A couple of studies are available evaluating the effects of the diet on the ruminal virome (Swain et al., 1996; Anderson et al., 2017). Swain et al. (1996) using pulse-field gel electrophoresis and two groups of sheep, one fed oaten and chaff-lucerne at a ratio of 70:30 and the other fed pasture, reported differences in the viral population between groups. They hypothesized that differences on viral population between dietary groups are due to the specific dietary regime and suggested that it is possible to manipulate the rate of bacterial lysis (Swain et al., 1996). Also, the authors observed a diurnal fluctuation in the viral population in response to feeding.

In a more recent study, Anderson et al. (2017) used a metagenomic approach, to evaluate the effect of diets varying in energy content, nutritional composition, and fiber source in beef steers. They demonstrated that virus population can change according to the diet and viruses can modulate the microbiome, impacting microbial metabolism.

**FIGURE 2:**
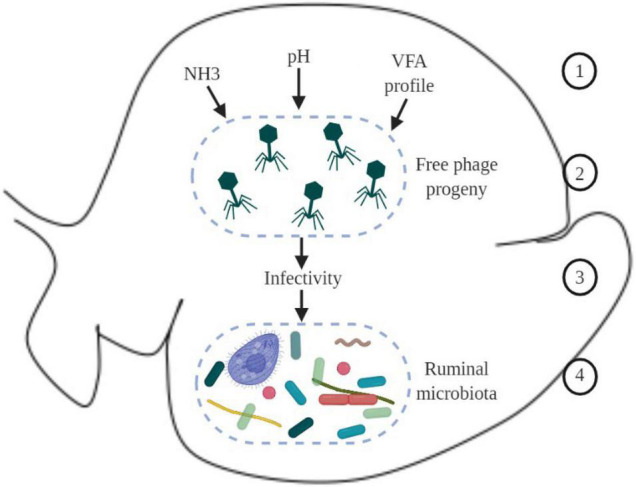
Schematic overview of hypothetical environmental factors in the ruminal environment that could affect virus dynamics and phage-host interaction. 1 – physicochemical parameters in the ruminal environment, such as ammonia (NH_3_), pH, and VFA (volatile fatty acids); 2 – new phages produced; 3 – physicochemical parameters in the environment can modulate infectivity of new phages; 4 – changes in the infectivity of phages can modulate the ruminal microbiome.

Based on knowledge from other fields, such as the marine environment, it can be speculated that when new free virions are produced in the rumen, physicochemical properties (such as pH, ammonia, volatile fatty acids, and other metabolites) can modulate viral activity, increasing or reducing their infectivity to the next host cell (Figure 2); however, to our knowledge this has not yet been shown in the rumen.

**FIGURE 3:**
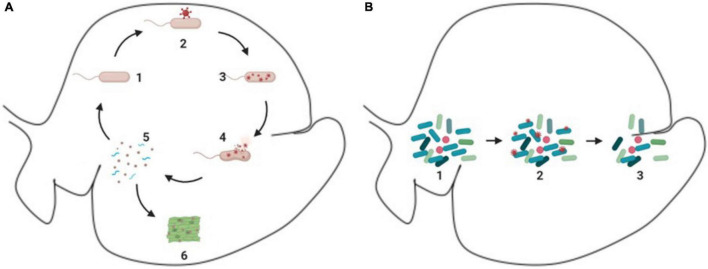
Schematic demonstration of phages and ruminal microorganisms. **(A)** Process of intra-ruminal recycling and enzymatic release. 1 – microorganism in the environment (e.g., rod like microorganism); 2 – attachment of phages (red circular structure) to microorganism; 3 – reproduction of phages (red circular structure) inside of the host cell; 4 – lysis of the host cell and release of virions (red circular structure) and cellular particles; 5 – cellular particles released (blue spiral like particles), which can be used as substrate to other microorganisms; 6 – enzymes released can act in the degradation of feedstuff. **(B)** Bloom of lytic cycle. 1 – microbiome with predominant species (dark blue rod like microorganisms); 2 – phages (small red circular particles) attachment to individuals of the predominant species (dark blue rod like microorganisms); 3-reduction of the predominant species (dark blue rod like microorganisms).

As discussed previously, viruses can infect ruminal microorganisms and many of these interactions between the viruses and microorganisms in the ruminal environment can generate a fragmentation of the host cell, which releases particles of the host cell, including proteins, nucleic acids, and cell wall fragments, which can be used by other organisms from the microbiome as source of nitrogen and energy, in a process referred as intra-ruminal recycling (Firkins et al., 1992; Hartinger et al., 2018) (Figure 3A). The microorganisms involved in the recycling process utilize substrates that are not primarily absorbed by the animal, using it as a source of nutrient to produce microbial biomass, and later on the microbial biomass produced can be digested, absorbed, and metabolized by the animal (Silva et al., 2019; Gnetegha Ayemele et al., 2020).

In addition, a recent study suggested that the bacterial host cell lysis can release enzymes as well, including those involved in carbohydrate fermentation (Solden et al., 2018). These enzymes can help in the feed degradation and enhance ruminal fermentation. These findings support the idea that phages in the rumen can influence microbial lysis and nutrient recycling.

Phages can drive the environment ecology by bloom of lytic activity, which is illustrated in Figure 3B, this process occurs in the rumen and was first presented by Swain et al. (1996). These lytic activities affect the dominant microbial population in the environment in a process named top-down or “kill the winner,” which is a theoretical model, in which a specific organism starts to dominate the ecosystem, this specific organism will likely be preyed upon by its natural viral predator, which will control the organism population, creating the opportunity for other organisms to emerge. However, this has not been experimentally demonstrated in the ruminal environment (Gilbert et al., 2020).

## The Prospectus of Phage Therapy in Ruminants

After the discovery of phages infecting bacterial cells and their potential to kill the host, Felix d’Herelle was the first to use phages as a treatment to reduce human infections (d’Herelle, 1917). This technology has continued to be developed since then and studies of biological control of several human infections have been carried out, bacterial diseases such as pneumonia (Dufour et al., 2019; Anand et al., 2020) and skin infections (Jault et al., 2019) are some examples of its application. In addition, studies using phage therapy to control antibiotic resistant organisms have been successfully used (Schooley et al., 2017).

According to Gilbert and Klieve (2015), in agriculture, phage therapy approaches for biological control have caught the attention of researchers. This shift from antibiotic use to the phage therapy is advantageous due to the fact that the isolation of a phage of interest is relatively simple, fast, and inexpensive, also, due to its high specificity of target, which avoids disturbances of local microbiome (Parasion et al., 2014).

Phage therapy has been studied as a strategy to reduce methane (Leahy et al., 2010; Morkhade et al., 2020). As ruminal fermentation leads to the eventual production of methane, it is thought that perturbations of microbial populations that contribute to this process would result in a reduction of methane. Methanogens act as a hydrogen sink, combining hydrogen with carbon dioxide to produce methane (Ungerfeld, 2020), and therefore targeting methanogens with phage activity to control their population and activity would lead to a reduction in methane production. Indeed, this was shown to be the case when archaea population is reduced, the activity of methane pathway is decreased and the hydrogen is redirected toward other metabolic pathways available in the rumen that are more beneficial, such as propionate production (Martin and Macy, 1985). According to Leahy et al. (2010), the discovery of a prophage with 69 phage-related proteins have been described from *Methanobrevibacter ruminantium*, including lytic enzymes which have a potential to be applied as a biocontrol agent for ruminal methanogens, such as Peir from the Methanobrevibacterium prophage φmru. These enzymes may potentially be used to reduce the archaea population in the rumen which could lead to reductions in methane emission and consequently less energy loss from feed.

Altermann et al. (2018) studied the viability of the production of phage-derived lytic enzymes, they used the viral enzyme PeiR from methanogen virus that has the capability to infect *Methanobrevibacter ruminantium*. They fused the gene of PeiR to polyhydroxyalkanoate synthase gene and added it in the genome of *Escherichia coli*. Translation of this gene produced a polyhydroxyakanoate with an active PeiR enzyme at the surface of this nanoparticle. Altermann et al. (2018) also reported that these nanoparticles were able to kill not only the original methanogen host strain cell but a wide range of other ruminal methanogen strains in an *in vitro* pure culture, reducing methane emission by up to 97%.

Another application of phage therapy is to revert cases of dysbiosis. According to Belizário and Napolitano (2015) and Gutiérrez and Domingo-Calap (2020), phage therapy can be used to reestablish the homeostasis of the host microbiome. Under dysbiosis, the homeostasis of the host microbiome is altered, which leads to an increase in pathogenic organisms and a reduction on symbiotic organisms (Palmela et al., 2018), using phage therapy, the organisms that are causing the dysbiosis can be targeted, leading to a reestablishment of homeostasis of the host microbiome.

Recently, a study was published testing an endolysin (LyJH307) to reduce *Streptococcus bovis*, which is a lactic acid-producing bacteria that is highly correlated to development of subacute ruminal acidosis (Kim et al., 2020). According to the authors, the viral molecule LyJH307 presented a potent lytic activity in a wide range of pH and temperature. Also, it was effective in the control of growth not only *S. bovis* isolated from rumen, but in in different groups of *S. bovis*, suggesting that this molecule have the potential to be used in the control of *S. bovis* which is one of the biggest contributors to the development of subacute ruminal acidosis.

## Use of Technology

For the scope of this article, only a brief summary of the technologies used in phages studies was included; however, a comprehensive publication on advances in technology for studying rumen virome has been recently published by Gilbert et al. (2020). The infection of bacteria by phages was first noted in the early 1900’s (Twort, 1915; D’Herelle, 1918), a few years later, EM was developed (Haguenau et al., 2003). Electron microscopy is a technique that enable researchers to visualize particles at a nanometer scale, providing a direct image of the subject of study (Richert-Pöggeler et al., 2019), in this case the viral particle. Electron microscopy enabled pioneer studies with focus on visualization and enumeration of ruminal phages (Hoogenraad et al., 1967; Paynter et al., 1969).

Early work using EM to study the ruminal environment, used ruminal fluid filtered through muslin (which is a type of cotton cloth) and stained for negative contrast using potassium phosphotungstate (Hoogenraad et al., 1967). The results of this study showed a large number of viral particles, either free or attached to different types of cells, especially icosahedral particles and tailed phages (Hoogenraad et al., 1967), although it was observed a large number of phages in the rumen, a quantitative estimation of phages was not made.

Following this pioneering study, a few other studies were published using similar methodologies to investigate ruminal phages from different species of ruminants, such as cattle (Paynter et al., 1969; Klieve and Bauchop, 1988), reindeer (Tarakanov, 1972), and sheep (Klieve and Bauchop, 1988). The morphology of the phages was studied in all of these studies, which reported up to 26 different types of phages and an estimation of viral particle counts ranging from 5 × 10^7^ to 1.4 × 10^8^ viral particles per mL of ruminal content of sheep and cattle (Table 1).

Isolation of phages from the ruminal environment was also carried out. Adams et al. (1966) successfully isolated phages from ruminal fluid and were able to demonstrate the concept of phage specificity. They reported that phages that were able to infect *Serratia* host strains from the rumen were unable to infect *Serratia* strains from other environments, such as soil, water, and sewage (Adams et al., 1966). Also, Hoogenraad et al. (1967) demonstrated with EM that phages in the rumen were different from phages from other environments. The isolation technique commonly used was culture-based, in which double-layer agar plates were used for detection of clearing zones within bacterial monolayers (Klieve, 2005). However, those culture-based methods tend to favor the isolation of phages undergoing lytic cycle (Gilbert et al., 2020), which were the most studies forms of phages.

This technology has been used to this date; however, morphological study of phages occasionally create issues such as misclassification due to similar morphological types (Ackermann, 2013) or even identify a false viral particle, such as in cases where cytoplasmic structures were identified as viral particles (Bullock et al., 2021). Also, compared to modern DNA techniques, EM underestimates the richness of viral species (Ross et al., 2013). Nevertheless, EM is still being used as a powerful tool to study the morphology of new isolated phages (Lima et al., 2019; Qin et al., 2019; Park et al., 2020).

The fast development of molecular biology methodologies has allowed rumen microbiologists to use those techniques in the study of ruminal phages (Orpin et al., 1988; Flint, 1997). Techniques based on genome length (such as electrophoresis and blotting techniques) and restriction enzyme mapping have been more commonly utilized (Gilbert et al., 2020). Klieve and Swain (1993), used intact genome lengths and pulsed field gel electrophoresis techniques to build a population profile and estimate phages count, which ranged from 3 × 10^9^ to 1.6 × 10^10^ viral particle per mL of ruminal content. The pattern observed was mainly of DNA length ranging from 30 to 200 kilo bases, which comprises many types of phages, including temperate ones.

With the advance of molecular biology techniques, total DNA sequencing (commonly known as shotgun metagenomics) (Ross et al., 2013; Anderson et al., 2017; Namonyo et al., 2018) has been used and reported as a good tool for the study of ruminal virome; however, metaproteomics (Solden et al., 2018) and metatranscriptomics (Hitch et al., 2019) have also been used. In shotgun sequencing reports, high throughput sequencing tool is used to sequence the total DNA of a sample, providing millions of reads which are assembled by bioinformatics tools allowing the researchers to identify DNA viruses and annotate the genome. However, the shotgun metagenomic does not include population of RNA viruses and metatranscriptomic is needed to capture this population.

Also, sequencing of DNA from isolated viruses is a powerful strategy to obtain the genomic information, functional prediction, and a better understanding of phages and its interaction with bacteria. Together, Gilbert et al. (2017) and Friedersdorff et al. (2020), reported the entire genome of 10 ruminal phages. According to Friedersdorff et al. (2020), sequenced phages were in a lytic life cycle; however, the functional genomic analysis enabled to infer that some of the phages had lysogeny-associated genes, which suggest that some of these phages could have a temperate life cycle as well. Gilbert et al. (2017), provided insights on how lytic phages interact with the host bacteria in the rumen environment.

The use of “*omics*” approaches, enable researchers to identify a large number species, Ross et al. (2013) reported an estimation of diversity of up to 27,000 species in the cow’s ruminal virome; however, the majority of these species still unknown. Namonyo et al. (2018) reported that more than 90% of the viral reads could not be identified, due to the limited availability of viral genomes in current databases, suggesting that a great amount of genomes are still unknown, creating opportunities to more identification studies. Also, *in silico* analysis of the viral genome, suggested that ruminal phages have glycosidic hydrolases, which could potentially increase the degradation of carbohydrates and consequently increase dietary energy efficiency (Anderson et al., 2017). Indeed “*omic*” approaches can help in the development of the understanding of the virome and its interactions; however, a great amount of variability of the estimated richness and small number of samples were observed (Table 2).

**TABLE 2:** Summary of published studies characterizing the ruminal virome using high throughput sequencing tools.

Host species	Richness	Method used	Number of animals	Feed used	Source
Cattle	17,993	N of unique viral contigs^a^	3	NA	Berg Miller et al. (2012)
Cattle	435,304	CatchAll (Allen et al., 2013)	13	6 kg of concentrate + *ad libitum* lucerne hay	Ross et al. (2013)
Buffalo	3,239	N of unique viral contigs^a^	1	Pasture	Parmar et al. (2016)
Cattle	∼1000	Chao1	5	Total mixed ration	Anderson et al. (2017)
Sheep and goat	179 and 1,456	N of unique viral contigs^a^	8 and 8	NA	Namonyo et al. (2018)
Moose	810	N of unique viral contigs^a^	1	Wild pasture	Solden et al. (2018)
Sheep	2,466	N of unique viral contigs^a^	20	Pelleted lucerne	Hitch et al. (2019)

*^a^Number of assembled contigs and identified as virus by homology to other known viruses.*

## Conclusion

Viruses are an important component of the ruminal environment and likely play roles in the ecology of the rumen; however, their activation mechanisms in the rumen remain unclear and their interactions with other components of the microbiome, diet, and physicochemical properties of the ruminal environment and subsequent effects on the health and production of livestock animals. The limited number of viral studies, lack of rumen-representative data, limited number of rumen isolates of phages, and very little information on mycophages and protozoal viruses creates a variety of opportunities for future studies. Also, very little is known about the effects of transient viruses in the gastrointestinal tract. An international effort to investigate phages should be developed, similar to the global effort employed to understand the bacterial population and their interactions with health of humans and animals.

Moreover, phages can be used as a powerful biotechnological tool in livestock production, they may have applications in areas such as pathogen control, gastrointestinal tract homeostasis regulators, methane emission reducers, and thus improve energy efficiency in the rumen. However, phages’ current applications in livestock production systems are still below their potential due to the lack of knowledge and research in the basic and applied ruminal virome.

## Author Contributions

RL and AF conceived the idea. RL collected literature data, created the tables and figures, and wrote the first draft of the manuscript. AF reviewed and edited the manuscript. Both authors approved the final version of the manuscript.

## Conflict of Interest

The authors declare that the research was conducted in the absence of any commercial or financial relationships that could be construed as a potential conflict of interest.

## Publisher’s Note

All claims expressed in this article are solely those of the authors and do not necessarily represent those of their affiliated organizations, or those of the publisher, the editors and the reviewers. Any product that may be evaluated in this article, or claim that may be made by its manufacturer, is not guaranteed or endorsed by the publisher.
